# Hungry to learn: the prevalence and effects of food insecurity on health behaviors and outcomes over time among a diverse sample of university freshmen

**DOI:** 10.1186/s12966-018-0647-7

**Published:** 2018-01-18

**Authors:** Meg Bruening, Irene van Woerden, Michael Todd, Melissa N. Laska

**Affiliations:** 1 College of Health Solutions, Arizona State University, 550 N 3rd Street, Phoenix, AZ 85004 USA; 2 College of Nursing and Health Innovation, Arizona State University, 500 N 3rd Street, Phoenix, AZ 85004 USA; 3 Division of Epidemiology and Community Health, University of Minnesota, 1300 South Second Street, Minneapolis, MN 55455 USA

## Abstract

**Background:**

To examine longitudinal associations between food insecurity (FI) and health behaviors/outcomes among a diverse sample of university freshmen**.**

**Methods:**

Freshman students (*n* = 1138; 65% female; 49% non-white) participating in the Social impact of Physical Activity and nutRition in College study completed surveys on health behaviors and had height/weight measured up to 4 times (T1-T4) in Arizona during 2015–2016. Structural equation models were estimated to determine if, after adjusting for covariates, FI predicted concurrent behaviors/outcomes and subsequent behaviors/outcomes. Analyses reported here were conducted in 2017.

**Results:**

The prevalence of FI was significantly higher at the end of each semester (35% and 36%, respectively) than at the start of the year (28%). Longitudinally, FI was not related to any health behaviors/outcomes at future time points. However, FI was significantly and inversely associated with concurrent breakfast consumption on most days of the week (OR = 0.67, 99% CI = 0.46, 0.99), daily evening meal consumption (OR = 0.55, 99% CI = 0.36, 0.86) healthy eating habits on campus (OR = 0.68, 99% CI = 0.46, 1.00), healthy physical activity habits on campus (OR = 0.66, 99% CI = 0.44, 1.00), and positively related to the likelihood of experiencing stress (OR = 1.69, 99% CI = 1.16, 2.46) and depressed mood (OR = 1.98, 99% CI = 1.34, 2.91).

**Conclusions:**

Compared with US prevalence rates, the sample FI prevalence was high. FI was related to poorer eating patterns, physical activity behaviors, and mental health, even after adjusting for prior levels of behavior.

## Background

The prevalence of food insecurity in the US has decreased slightly over time [1]; however, it is still high (15%) and some populations are at greater risk than others. Numerous recent cross-sectional studies have examined the high rates of food insecurity among students attending post-secondary institutions both in the US and internationally [2] . These studies have identified rates of food insecurity ranging from 12.5% to 84% [3–5], with a systematic review calculating an average food insecurity among students at 42% [2]. Post-secondary students who report food insecurity are more likely to be students of color [5, 6], financially independent [4, 7, 8], younger and/or students with families [3, 4, 8]. Compared with food secure students, food insecure students are more likely to be at risk for poor health and report lower fruit and vegetable consumption [3], less frequent breakfast intake [9], and worse mental health outcomes [9]. In addition, food insecurity appears to be related to poor academic outcomes. For example, Maroto et al. reported food insecure students were less able to concentrate than food secure students [7, 10] . Several studies have found a significant association between food insecurity status and lower grade point averages [5, 7]. In the US, the Supplemental Nutrition Assistance Program (SNAP—formally known as the Food Stamps Program) provides an important safety net for those with food insecurity; however, for university students, SNAP has additional requirements that prohibit some students’ participation [11]. In order for university students to be eligible for SNAP, they must work a minimum of 20 h per week, have dependents between the ages of 5–12 and not have childcare, participate in work-study programs, or have other waivers https://www.fns.usda.gov/snap/students) [11]. Despite evidence suggesting poorer health and academic outcomes for food insecure university students, to date no study has examined the effects of food insecurity on these outcomes over time. Cross-sectional associations between food insecurity and various outcomes cannot address temporality and may be inflated by unmeasured residual confounding.

University campus food environments can contribute to poor eating behaviors with all-you-can-eat dining halls, limited hours of operation and high prices at healthy dining eateries, and limited access to grocery stores [12–14]. Future research should consider these factors as they relate to food insecurity. Studying food insecurity over time among post-secondary freshmen students is of particular interest. Freshmen, especially those living away from parents and guardians for the first time undergo significant social, emotional, and behavioral transitions [15, 16], being independent for the first time in their emerging adult lives. These students report higher levels of stress [17, 18], poorer eating behaviors [19], and higher rates of weight gain [20, 21], as compared with older students. Given that some studies have reported that younger students are at greater risk for food insecurity [4], the additive effects of co-occurring developmental transitions and increased risk of food insecurity may have significant impact on health and academic performance over time. Longitudinal studies can aid in understanding how to prevent food insecurity and promote healthy behaviors and development.

## Methods

### Study sample

This manuscript is based on a secondary analysis of a large, NIH-funded study, SPARC (Social impact of Physical Activity and nutRition in College), aimed at assessing the nutrition, physical activity behaviors and weight outcomes of college freshmen. College freshmen living in six residence halls on three campuses of a single metropolitan university during the fall and spring of the 2015–2016 academic year were recruited to participate primarily via floor meetings held at the residence halls, shortly after students moved into the residence halls. At the institution where these data were collected, students are required to purchase a meal plan if they live in the residence halls. However, this meal plan can cover as few as 8 meals per week. In addition, under certain conditions, students are allowed to opt out of the meal plan purchasing requirement. Informed written consent from all participants was obtained. For this study, we analyzed data from survey measures and measured anthropometrics obtained during August 2015, November 2015, January 2016 and April 2016 (Times 1, 2, 3, and 4, respectively). For each completed assessment, participants earned incremental monetary awards (up to $110) and additional earned incentives (e.g., study branded water bottles, t-shirts, Frisbees, ear buds, tote bags). More details about the study design and protocols have been published elsewhere [22]. All study protocols were approved by the Arizona State University Institutional Review Board.

#### Measures

At each wave of data collection, anthropometric variables were measured, and participants completed web-based surveys. Surveys took 20–30 min to complete and assessed self-reported eating, alcohol, physical activity, sleep, and mental health.

#### Food insecurity

The USDA six-item food security short form was used to assess food security status [23]. To better examine temporal effects of food insecurity for individual college students, we altered the scale to measure food security over the past month instead of the past year. The survey module for this study included the following items: “Is this statement true?: ‘The food that I bought just didn’t last, and I didn’t have money to get more’”; “I couldn’t afford to eat balanced meals’”; “In the past 1 month, did you ever cut the size of your meals or skip meals because there wasn’t enough money for food?”; “In the past 1 month, did you ever eat less than you felt you should because there wasn’t enough money for food?”; and “In the past 1 month, were you ever hungry but didn’t eat because there was not enough money for food?” Any participant responding affirmatively to 2 or more questions in a given assessment was categorized as being food insecure at that time point. At Time 1, the assessment of 1 month prior food security status would have assessed food insecurity prior to moving to the residence hall or just as students had started the semester.

#### Body composition

Participant weight and height were measured by trained research assistants. Weight was recorded to the nearest 0.1 kg using portable Seca™ flat scales (models 874 or 869) and height to the nearest 0.1 cm using portable Seca™ stadiometers (model 217). Two measurements were recorded and averaged for weight and height; if the difference of the two measurements were off by more than 0.5 kg or 0.5 cm, respectively, additional measurements were taken until the difference between pairs of measurements less than 0.5 kg or 0.5 cm. BMI was calculated, and participants with a BMI ≥ 25 kg/m^2^ were classified as overweight/obese.

#### Eating behaviors

The validated 26-item Dietary Screener Questionnaire (DSQ) [24] was used to assess consumption of major food groups (e.g., fruits and vegetables, high fat foods, sugar sweetened beverages) over time. In addition, participants were asked "In the past 7 days, how often did you eat the following: breakfast, evening meals, and fast food " [25]. Response options ranged from never to 7 days and, based on the distribution of the responses, were dichotomized (see Table 2). The number of servings of fruit/vegetables participants consumed over the past week was determined by asking participants "Thinking back over the past week, how many servings of fruit did you usually eat on a typical day? (A serving is half a cup of fruit or 100% fruit juice or a medium piece of fruit)" and "Thinking back over the past week, how many servings of vegetables did you usually eat on a typical day? (A serving is half a cup of cooked vegetables or one cup of raw vegetables)" [26]. Fruit and vegetable responses were summed and then dichotomized to 4 or more servings of fruit/vegetables per day vs less. To examine perceived eating habits participants were asked "How would you rate your eating habits: on campus and off campus?". Response options were on a four-point scale from very unhealthy to very healthy and were dichotomized to unhealthy vs healthy.

#### Alcohol behaviors

To determine alcohol behaviors, participants were first asked, “Have you ever drank alcohol?” Participants who reported any lifetime drinking were asked about their binge drinking habits (5 drinks or more per drinking occasion for males; 4 drinks or more per occasion for females) [27]. Responses were converted to presence (yes/no) of binge drinking.

#### Physical activity and leisure-time behaviors

Vigorous, moderate, and light PA were assessed with the Godin-Shepard PA assessment [28], which assesses usual PA: “In a usual week, how many hours do you spend doing the following activities: Strenuous exercise (heart beats rapidly)?; Moderate exercise (not exhausting)?; Mild exercise (little effort)?” Response options ranged from none to more than 6 h per week. From this measure, a dichotomous measure of time spent in moderate-to-vigorous PA (MVPA) per day was created (≥ 30 min/day vs. < 30 min/day) Participants also rated their perception of the healthfulness of their physical activity behaviors on and off campus [29]. Response options were a four-point scale from very unhealthy to very healthy and were recoded to unhealthy vs. healthy. Sedentary activities are assessed with the question: “Yesterday, how much time did you spend in front of a screen (excluding time in class and being physically active)?” [30]. Response options ranged from none to more than six hours and, based on the distribution of values observed in the sample, were recoded to ≥150 mins/day vs. less.

#### Mental health

Perceived stress during the past month was examined using four questions adapted from Cohen [31]. Items asked “How often in the past 1 month have you felt”: “Unable to control important things in your life?”, “Confident about your ability to handle your personal problems?”, “Things were going your way?”, and “Difficulties were piling up so high that you could not overcome them?” Past month depressed mood was examined using six questions adapted from American College Health Association survey (2013) [32] asking “How often in the past 1 month have you felt”: “Things were hopeless”, “Overwhelmed by all you had to do”, “Very lonely”, “Very sad”, “So depressed it was difficult to function”, and “Overwhelming anxiety”. Response options for perceived stress and depressed mood items ranged from 1 to 4 (never, rarely, sometimes, and often). Scores on perceived stress items were summed to create an overall perceived stress score. Any stress score higher than 8 were classified as high stress. Scores on depressed mood items were averaged to create an overall depressed mood score. Any score higher than 2 was classified as high depression. To assess anxiety participants were asked “In the past 12 months have you been told by a doctor or health professional that you have anxiety?”, with response options of “no”, “yes, diagnosed and treated”, “yes, diagnosed”, and “yes, treated” [33]. A dichotomous indicator was created such that any “yes” response indicated anxiety was present.

#### Sociodemographics

Data on participants’ gender, race/ethnicity, Pell grant status, and current residence hall were collected. Participants were recruited from 6 residence halls (4 residence halls from Campus A and 1 from Campus B, and 1 residence Hall from Campus C). Based on the distribution of participation across residence halls and campuses, participants were ultimately classified into two categories Residence hall A and Residence halls B. Race/ethnicity was included in the analyses as a potential confounder. Every participant was asked “how do you usually describe yourself? (check all that apply)” with the response options “White”, “Black or African American”, “Hispanic or Latino/a”, “Asian or Pacific Islander”, “American Indian or Alaska Native”, and “Some other race”. Due to low numbers in some categories, race/ethnicity was categorized as Hispanic, non-Hispanic White, non-Hispanic Black, or other. Every participant was asked if he or she was a recipient of a grant from the Federal Pell Grant program, which provides financial aid for low-income university enrollees. Responses for Pell grant status were coded as yes or no.

### Statistical analysis

**Fig. 1:**
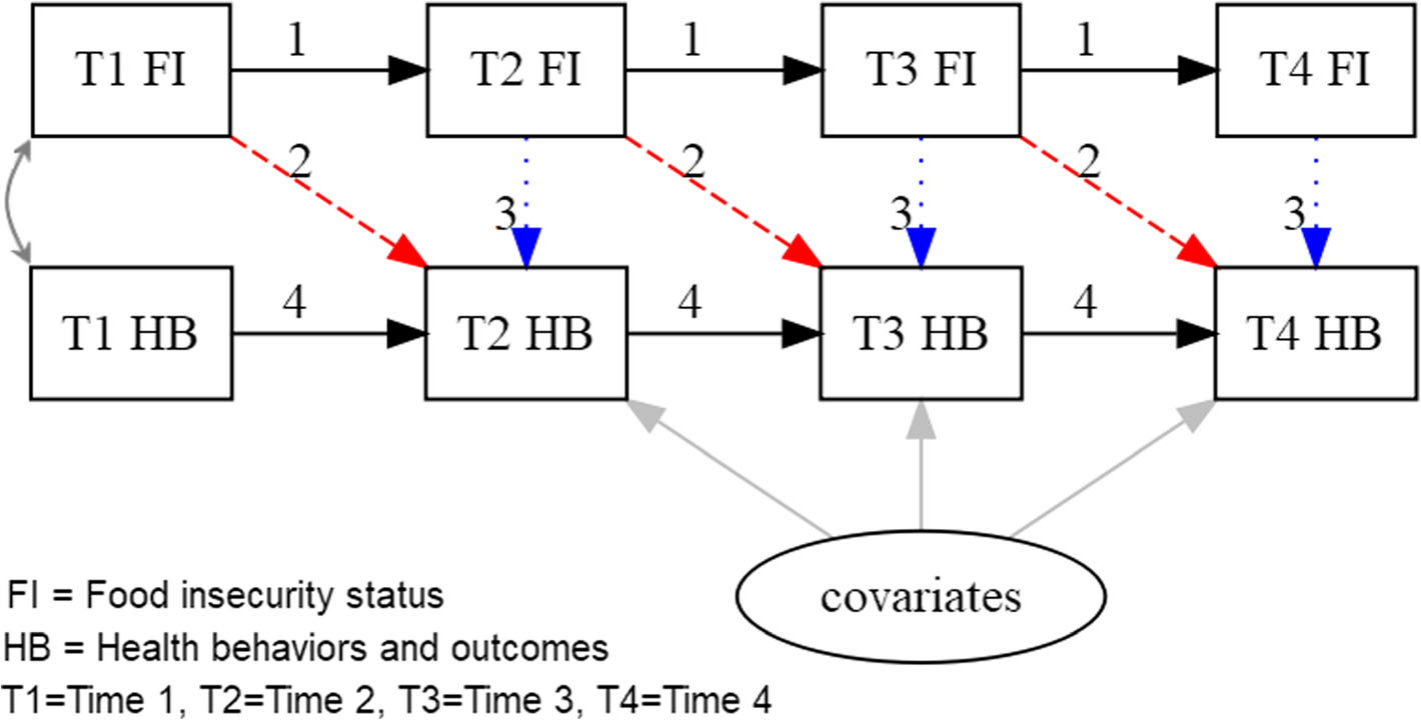
Structural equation models used to determine how previous and concurrent food insecurity (FI) were related to the reported health behaviors and outcomes (HB) across times 1 (T1), 2 (T2), 3 (T3), and 4 (T4) among college freshmen in Phoenix, AZ metro during 2015–2016 academic year. T1 food insecurity and T1 health behavior and outcomes were set to covary. Paths to T2, T3, and T4 health behavior and outcomes were adjusted for gender, race/ethnicity, Pell grant status, and residence hall

The analysis compares 1 residence hall with the highest response to the other residence halls (*n* = 5) due to sample size constraints. Descriptive analyses were used to examine the distribution of key variables. Pairwise chi-square tests were used to examine the prevalence of food insecurity over time, and to determine how food insecurity was associated with the health behaviors and outcomes for each of the four time points. While there was significant dropout over time, food security status was not predictive of participation (data not shown). Dropout was predicted by sex and campus (females and participants from Campus A were more likely to remain in the study as compared to males and participants from Campus B and C); no difference in participation by race/ethnicity or Pell grant status was observed. Repeated measures logistic regression generalized estimating equations (GEE) models were used to determine if the prevalence of food insecurity differed significantly across time points, adjusting for gender, race/ethnicity, Pell grant status, and residence hall group. Structural equation path models (SEMs) were used to determine how previous and concurrent food insecurity were simultaneously associated with each health behavior and outcome at each time point. The SEM used for this analysis adjusted for the observed outcome at the preceding time point (for T2-T4), food insecurity at the preceding time point (for T2-T4), and food insecurity at the current time point (for T1-T4) (e.g. T4 weight status was predicted by T3 weight status, T3 food security status, and T4 food security status). See Fig. 1 for an illustration of the model. For each health behavior and outcome, four sets of like model paths (labeled 1–4 in Fig. 1, e.g., all prospective prediction paths, labeled as 2) were examined to determine if like path estimates differed significantly from each other. Tests of relative model fit showed that models with like paths freely estimated fit no better than models with all like paths constrained to be equal over time (i.e., the most parsimonious model; *p* > 0.01, results summarized below). The association between food insecurity and each outcome at Time 1 was estimated as a covariance rather than as a regression (“causal”) path. Associations with health behaviors and outcomes at Times 2–4 were adjusted for gender, race/ethnicity, Pell grant status, and residence hall group. All analyses reported here were conducted in 2017 using the R (v 3.3.2) and Mplus (v 7.4) statistical packages. Due to multiple tests, statistical significance for model fit was determined at *p* < 0.05 and in the SEMs was determined at *p* ≤ 0.01.

## Results

At baseline (Time 1) the sample consisted of 1138 freshmen (65% female, 49% non-white; Table 1). The sample consisted of 555, 428, and 400 freshmen at times 2, 3, and 4, respectively. Bivariate analyses showed Pell grant recipients were significantly more likely to report food insecurity at Time 1 (*p* ≤ 0.01) than non-Pell grant recipients; differences by residence hall groups were also found at Times 2 (*p* ≤ 0.01) and 3 (*p* ≤ 0.01). No differences were observed between gender, or race/ethnicity, and food insecurity at any of the time points.

**Table 1:** Frequencies for key demographics by food secure (FS) and food insecurity (FI) status among college freshmen across time points

	Time 1			Time 2			Time 3			Time 4		
	Total	FS	FI	Total	FS	FI	Total	FS	FI	Total	FS	FI
Total, n(%)	1138	815 (72)	323 (28)	555	360 (65)	195 (35)	428	292 (68)	136 (32)	400	258 (64)	142 (36)
Gender, n(%)												
Female	741 (65)	540 (66)	201 (62)	402 (72)	261 (72)	141 (72)	315 (74)	222 (76)	93 (68)	291 (73)	191 (74)	100 (70)
Male	397 (35)	275 (34)	122 (38)	153 (28)	99 (28)	54 (28)	113 (26)	70 (24)	43 (32)	109 (27)	67 (26)	42 (30)
Race/ethnicity, n(%)												
White	576 (51)	431 (53)	145 (45)	274 (49)	183 (51)	91 (47)	200 (47)	142 (49)	58 (43)	180 (45)	123 (48)	57 (40)
Hispanic	277 (24)	181 (22)	96 (30)	129 (23)	83 (23)	46 (24)	109 (25)	69 (24)	40 (29)	103 (26)	56 (22)	47 (33)
Black	104 (9)	77 (9)	27 (8)	61 (11)	33 (9)	28 (14)	47 (11)	33 (11)	14 (10)	48 (12)	32 (12)	16 (11)
Other	181 (16)	126 (15)	55 (17)	91 (16)	61 (17)	30 (15)	72 (17)	48 (16)	24 (18)	69 (17)	47 (18)	22 (15)
Pell Grant status, n(%)^T1^												
No	766 (67)	575 (71)	191 (59)	360 (65)	247 (69)	113 (58)	279 (65)	195 (67)	84 (62)	252 (63)	173 (67)	79 (56)
Yes	372 (33)	240 (29)	132 (41)	195 (35)	113 (31)	82 (42)	149 (35)	97 (33)	52 (38)	148 (37)	85 (33)	63 (44)
Residence hall, n(%)^B,T2, T3^												
A	681 (60)	473 (58)	208 (64)	267 (48)	153 (42)	114 (58)	197 (46)	121 (41)	76 (56)	195 (49)	116 (45)	79 (56)
B	457 (40)	342 (42)	115 (36)	288 (52)	207 (57)	81 (42)	231 (54)	171 (59)	60 (44)	205 (51)	142 (55)	63 (44)

^T1, T2, T3^chi-square test significant at *p* ≤ 0.01 at Times 1,2, and 3. No significant bivariate associations were observed at Time 4 [T4]

^B^Residence hall indicates residents from one campus. Residence hall B indicates all other resident participants

Bivariate analyses showed the prevalence of food insecurity was significantly higher at the end of the first semester (Time 2; 35%, *p* ≤ 0.01) and end of the second semester (Time 4; 36% *p* ≤ 0.01) when compared with the start of the first semester (Time 1; 28%). Even after adjusting for demographics, the odds of food insecurity were significantly higher at Time 2 (OR = 1.5, 99% CI = 1.1, 2.0), and Time 4 (OR = 1.5, 99% CI = 1.1, 2.0) than at Time 1. There was no difference in the odds of food insecurity at the beginning of the second semester (Time 3) compared with Time 1 (OR = 1.3, 99% CI = 0.9, 1.7).

At all four time points, bivariate analyses showed that food insecurity was significantly higher among participants who concurrently reported not regularly consuming an evening meal, high levels of stress, and high levels of depressed mood (*p* ≤ 0.01; Table 2). At two to three time points, bivariate analyses showed food insecurity was significantly higher among participants who did not regularly consume breakfast, who had unhealthy eating habits on campus, who did not obtain enough sleep, who felt tired throughout the day, and those reporting anxiety (*p* ≤ 0.01; Table 2). For example, among students who were food insecure at Time 1, 46% consumed breakfast 4 or more times/week; this finding was significantly less than the 63% of food secure students at Time 1 who consumed breakfast 4 or more times/week.

**Table 2:** Frequencies of reported behaviors and outcomes by food security status among college freshmen across time points

		Time 1, n(%)		Time 2, n(%)		Time 3, n(%)		Time 4, n(%)	
Variable		FS^a^	FI^a^	FS^a^	FI^a^	FS^a^	FI^a^	FS^a^	FI^a^
Weight status									
Overweight/obese	Yes	242 (30)	108 (35)	108 (36)	67 (42)	89 (35)	42 (35)	73 (34)	50 (42)
Eating behaviors									
Breakfast consumption^T1,T2,T4^	≥4 times /week	510 (63)	148 (46)	196 (55)	70 (36)	187 (64)	69 (51)	138 (53)	49 (35)
Evening meal consumption^T1,T2,T3, T4^	7 times /week	709 (88)	236 (74)	299 (84)	137 (71)	257 (88)	105 (78)	219 (85)	92 (66)
Fast food consumption	≥2 times /week	209 (26)	100 (31)	59 (16)	45 (23)	68 (23)	40 (30)	56 (22)	42 (30)
Fruit/vegetable consumption^T1^	≥4 servings /day	349 (43)	109 (34)	142 (40)	57 (29)	131 (45)	60 (44)	117 (45)	49 (35)
DSQ^b^ – fruits and vegetables	> 2 cups/day	473 (58)	180 (56)	184 (52)	79 (42)	167 (58)	69 (51)	124 (48)	57 (41)
DSQ^b^ - dairy	> 1.5 cups/day	488 (60)	188 (59)	186 (52)	89 (47)	117 (40)	58 (43)	115 (45)	58 (42)
DSQ^b^ – added sugars	> 16 tsp./day	420 (52)	163 (51)	156 (44)	86 (46)	102 (35)	53 (39)	102 (40)	49 (35)
DSQ^b^ – sugar sweetened beverages	> 6 tsp./day	494 (61)	202 (63)	209 (58)	113 (59)	136 (47)	68 (50)	140 (54)	76 (54)
DSQ^b^ – whole grains	>.65 oz./day	437 (54)	147 (46)	162 (46)	76 (40)	138 (48)	63 (47)	113 (44)	69 (50)
DSQ^b^ - fiber	> 15 g/day	419 (52)	166 (53)	156 (44)	61 (33)	120 (42)	60 (45)	103 (40)	50 (36)
DSQ^b^ - calcium	> 900 mg/day	522 (65)	185 (59)	184 (52)	94 (51)	125 (44)	65 (49)	112 (44)	65 (47)
Eating habits on campus^T1,T2,T3,^	Healthy	547 (67)	184 (57)	227 (63)	87 (46)	201 (69)	66 (49)	158 (61)	74 (52)
Eating habits off campus	Healthy	497 (62)	173 (55)	223 (62)	105 (55)	179 (62)	76 (56)	159 (62)	79 (57)
Alcohol behaviors									
Drink alcohol	Ever	575 (71)	238 (74)	224 (62)	138 (72)	165 (57)	88 (65)	148 (57)	87 (62)
Binge drinking	Yes	209 (37)	98 (41)	85 (38)	69 (50)	63 (39)	38 (43)	56 (38)	35 (40)
Physical activity (PA) and leisure time behaviors									
Moderate-vigorous PA	> 30 mins/day	539 (67)	197 (62)	196 (55)	95 (50)	160 (55)	73 (54)	147 (58)	76 (55)
PA habits on campus^T2^	Healthy	588 (73)	208 (65)	219 (62)	94 (49)	194 (67)	82 (62)	166 (65)	81 (57)
PA habits off campus^T1^	Healthy	532 (66)	172 (54)	206 (58)	88 (47)	159 (55)	64 (48)	149 (58)	66 (47)
Screen time	≥150 mins/day	531 (66)	191 (60)	278 (78)	143 (75)	209 (72)	89 (66)	186 (73)	93 (66)
Sleep quality									
Enough sleep^T1, T3^	≥5 times /week	280 (35)	62 (21)	90 (25)	30 (16)	112 (39)	32 (24)	56 (22)	18 (13)
Wake up too early	≥1 times /week	157 (20)	84 (27)	48 (13)	37 (20)	58 (20)	29 (21)	53 (21)	27 (19)
Feel tired during the day^T2, T3^	≥4 times /week	317 (40)	149 (47)	174 (49)	115 (61)	108 (37)	71 (53)	137 (54)	83 (60)
Mental health outcomes									
Perceived stress^T1, T2, T3, T4^	High ^c^	338 (41)	193 (60)	168 (47)	131 (67)	115 (39)	81 (60)	117 (45)	94 (66)
Depressed mood^T1, T2, T3, T4^	High^d^	294 (36)	189 (59)	141 (39)	129 (66)	110 (38)	71 (52)	104 (40)	95 (67)
Anxiety ^T1, T2^	Diagnosed or Treated	93 (11)	67 (21)	30 (8)	38 (19)	30 (10)	26 (19)	28 (11)	23 (16)

^T1, T2, T3,^T4chi-square test significant at *p* ≤ 0.01 at Times 1,2,3, and 4, respectively

^a^FS = food secure. FI = food insecure

^b^DSQ = Dietary Screener Questionnaire

^c^Perceived stress based on a 4-question, 4-point Likert scale; High was defined as > 8

^d^Depressed mood based on a 6-question, 4-point Likert scale; High was defined as > 2

Of the 104 tests of relative model fit comparing a model with sets of like paths freely estimated across time points to a corresponding model with like paths set to be equal, seven yielded *p*-values < 0.05, but > 0.01. The seven tests yielding *p*-values < 0.05 were as follows: tests for equality of paths for [1] prospective prediction (labeled as 2 in Fig. 1; *p* = 0.040) and [2] concurrent prediction (labeled as 3 in Fig. 1; *p* = 0.045) of depressed mood from FI and tests for equality of autoregressive paths (labeled as 4 in Fig. 1) for [3] overweight/obesity status (*p* = 0.033), [4] MVPA (*p* = 0.024), [5] on-campus PA habits (*p* = 0.014), [6] off-campus PA habits (*p* = 0.048), and [7] screen time (*p* = 0.033).

SEM results (Table 3) showed that when the concurrent (unlagged) association between food insecurity and the health behavior/outcome was accounted for at each wave (e.g., path from Time 1 food insecurity to Time 1 depressed mood), food insecurity at a given wave did not significantly predict the health behavior/outcome at the subsequent wave (e.g., path from Time 1 food insecurity to Time 2 depressed mood was not significant). Concurrent food insecurity, however, was significantly associated with lower odds of frequent breakfast consumption (OR = 0.67, 99% CI = 0.46–0.99), frequent evening meal consumption (OR = 0.55, 99% CI = 0.36, 0.86), healthy eating habits on campus (OR = 0.68, 99% CI = 0.46, 1.00), and healthy PA habits on campus (OR = 0.66, 99% CI = 0.44, 1.00). Concurrent food insecurity was also significantly associated with higher odds of stress (OR = 1.69, 99% CI = 1.16, 2.46), and depressed mood (OR = 1.98, 99% CI = 1.34, 2.91). For example, a students’ reported food insecurity at Time 1 was not significantly associated with a shift in their frequency of breakfast consumption at Time 2 (OR = 1.03, 99% CI = 0.68, 1.54); however, students’ reports of food insecurity were significantly associated with their concurrent breakfast consumption frequency, even after controlling for breakfast frequency at the previous time point (OR = 0.67, 99% CI = 0.46, 0.99).

**Table 3:** Odds ratios (ORs) and 99% confidence intervals from structural equation models with prediction of reported health behaviors and outcomes from previous and concurrent food insecurity

		Previous Food Insecurity		Concurrent Food Insecurity	
		OR	99% CI	OR	99% CI
Weight status					
Overweight/obese	Yes	0.56	(0.21,1.45)	1.68	(0.69,4.12)
Eating behaviors					
Breakfast consumption	≥4 times /week	1.03	(0.68,1.54)	0.67	(0.46,0.99)*
Evening meal consumption	7 times /week	0.93	(0.58,1.49)	0.55	(0.36,0.86)*
Fast food consumption	≥2 times /week	1.06	(0.68,1.65)	1.25	(0.83,1.88)
Fruit/vegetable consumption	≥ 4 servings/day	1.08	(0.72,1.62)	0.86	(0.58,1.25)
DSQ^a^ – fruits and vegetables	> 2 cups/day	1.11	(0.73,1.67)	0.76	(0.52,1.12)
DSQ^a^ - dairy	> 1.5 cups/day	1.27	(0.84,1.92)	0.81	(0.55,1.19)
DSQ^a^ – added sugars	> 16 tsp./day	1.10	(0.73,1.67)	0.91	(0.61,1.34)
DSQ^a^ – sugar sweetened beverages	> 6 tsp./day	0.95	(0.63,1.45)	0.90	(0.60,1.34)
DSQ^a^ – whole grains	>.65 oz./day	1.00	(0.68,1.46)	1.07	(0.74,1.53)
DSQ^a^ - fiber	> 15 g/day	1.05	(0.68,1.63)	0.88	(0.58,1.34)
DSQ^a^ - calcium	> 900 mg/day	1.21	(0.77,1.90)	0.98	(0.64,1.49)
Eating habits on campus	Healthy	0.84	(0.56,1.27)	0.68	(0.46,1.00)*
Eating habits off campus	Healthy	0.83	(0.55,1.25)	0.87	(0.59,1.28)
Alcohol behaviors					
Drink alcohol	Yes	1.09	(0.66,1.83)	1.27	(0.78,2.08)
Binge drinking	Yes	0.94	(0.57,1.56)	1.33	(0.83,2.13)
Physical activity (PA) and leisure time behaviors					
Moderate-vigorous PA	> 30 mins/day	0.94	(0.63,1.40)	0.95	(0.65,1.38)
PA habits on campus	Healthy	1.16	(0.75,1.79)	0.66	(0.44,1.00)*
PA habits off campus	Healthy	0.99	(0.65,1.50)	0.68	(0.46,1.01)
Screen time	> 195 mins/day	1.36	(0.88, 2.10)	0.76	(0.51, 1.13)
Sleep quality					
Enough sleep	≥5 times /week	0.72	(0.45,1.14)	0.69	(0.45,1.07)
Wake up too early	≥1 times /week	1.17	(0.74,1.87)	1.00	(0.64,1.57)
Feel tired during the day	≥4 times /week	1.14	(0.78,1.68)	1.39	(0.97,1.98)
Mental health outcomes					
Perceived stress	High^b^	1.42	(0.95,2.13)	1.69	(1.16,2.46)*
Depressed mood	High^c^	1.14	(0.75,1.74)	1.98	(1.34,2.91)*
Anxiety	Diagnosed or Treated	2.06	(0.92,4.62)	1.20	(0.55,2.59)

^a^DSQ—Dietary screener questionnaire

^b^Past month perceived stress score computed as sum of responses to 4 items with a 4-point response scale; High was defined as score > 8

^c^Past month depressed mood score computed as sum of responses to 6 items with a 4-point response scale; High was defined as score > 2

*indicates statistical significance at *p* ≤ 0.01

## Discussion

The purpose of this study was to examine how food insecurity is related to health behaviors and outcomes over time among college freshmen. We used structural equation path modeling to examine both prior food insecurity and concurrent food insecurity. The structural equation path model analyses showed that the paths from concurrent food insecurity were more strongly associated with health behaviors/outcomes than the paths from previous food insecurity, suggesting that at least in this population of university students, short-term effects of food insecurity on health outcomes are more notable. Meal patterns, perceptions of healthful eating, physical activity on campus, and mental health, were related to concurrent food insecurity. However, other dietary behaviors, weight status, alcohol consumption, and moderate-vigorous physical activity were not related to prior or concurrent food insecurity.

Given cross-sectional findings between food insecurity and dietary quality that have been previously reported in the literature [3, 9], we were surprised to not observe differences by food security status by dietary quality over time. It is possible that the dietary screening questionnaire used in this study was not sensitive enough to differentiate overall nutritional intake of the participants. It may also be possible that despite food insecurity status, the diets of this population are so poor [19] that differences in intake were not able to be detected. Future research is needed to understand how diet is impacted, if at all for students struggling with access to food.

Awareness of the prevalence of food insecurity on college campuses has grown in recent years [2, 34], but this is the first longitudinal study to examine the effects of food insecurity among college students. More research is needed to replicate and better understand these associations. Investigators may want to consider more frequent assessments of food insecurity among college students, as it may fluctuate with a greater frequency than what could be captured in the current study.

As reported in other studies [2–5], the prevalence of food insecurity was high among this sample, with over 25% reporting food insecurity at each of the four time points examined. The literature on college-based food insecurity suggests an average campus-based prevalence of food insecurity of approximately 40% [2] . Prevalence rates of food insecurity found in the current study are similar to those for a smaller pilot study at the same institution a year prior [9]. We observed increased reports in the prevalence of food insecurity at the end of the fall and spring semesters. These are times when students may have run out of food provided by parents [14], have exhausted dining hall meal allotments, and/or have experienced higher stress due to demands of final exams and projects. These findings also suggest extra outreach to students in need may be helpful at the end of each semester, particularly as food insecurity at the end of the semester may have a significant impact on academic success.

Overwhelmingly, based on this study, the lasting effects of food insecurity are not clear, but there appear to be adverse issues occurring concurrently with food insecurity that need to be simultaneously addressed [2]. More research is needed to better understand fluctuations in reports of food insecurity over time and how students cope with lack of consistent access to food. It is not clear if the struggle to access to food varies differentially for certain populations of students. Research is needed to identify and explain the causal mechanisms for students moving in and out of food insecurity, including other external factors. Students may use unhealthy coping strategies to supplement their income (e.g., meal skipping). The primary means by which post-secondary institutions are addressing food insecurity is through campus food pantries [2], or sites that provide free food, often procured by donation, to people in need. Food pantries can range in size of a closet to a small store. To date, there are over 500 food pantries on US college campuses [2, 34]. While food pantries may not address the root causes of food insecurity among students, given the current findings, food pantries may be an appropriate intervention to help those students with short-term, acute food insecurity. Unfortunately, no research exists on the effectiveness of college food pantries in addressing food insecurity, or the characteristics of these pantries, such as their longevity on campus, their student reach, the frequency with which they are offered and/or the types of foods they provide.

One particularly concerning finding, similar to previous findings in other settings, was that food insecurity was associated with mental health outcomes. Concurrent FI was associated with nearly two times higher the odds of experiencing high levels of stress and depressed mood. The mechanism for the relationship between food insecurity and mental and emotional health is likely bi-directional [2]. Screening tools have been developed to quickly assess food insecurity. Mental health clinics and other student resources centers may want to consider screening for food insecurity using brief assessment tools [35, 36], particularly during stressful times during the school year. Likewise, at sites on campus where food insecurity is being directly addressed (e.g., food pantries), referrals to health services may be warranted.

### Study strengths and weaknesses

This study fills an important gap in the literature, providing a view on the longitudinal nature of food insecurity. We were able to examine food insecurity at four time points, providing novel information on time-varying effects of food insecurity. Limitations of this study include limited generalizability, as we only studied a sample of freshmen at one institution. Results may vary by year of enrollment, region in the US and globally, and campus characteristics. Despite this limitation, one strength of the study is that the sample in this study was relatively large and diverse in terms of race/ethnicity. Almost all the measures were self-reported; as such, these measures are subject to social desirability and recall biases. Finally, we were limited to an examination of prospective relationships of food insecurity with health behaviors and outcomes. The nature of the study design precludes examination of the causes of food insecurity.

## Conclusions

Food insecurity among college students is highly prevalent, resulting in a major public health problem that adversely affects health outcomes. The findings in this study suggest that it is important to identify food insecurity to promote concurrent healthy behaviors. When students are struggling, they may be coping with several challenges simultaneously; thus, multifaceted intervention strategies addressing a variety of issues may be needed. As college enrollment is becoming more diverse and students have varying needs, public health professionals should work with universities to systematically screen for food insecurity. More public health research is needed on how to prevent and address food insecurity on and before students come to university.
